# Extreme Electron‐Photon Interaction in Disordered Perovskites

**DOI:** 10.1002/advs.202405709

**Published:** 2024-10-02

**Authors:** Sergey S. Kharintsev, Elina I. Battalova, Ivan A. Matchenya, Albert G. Nasibulin, Alexander A. Marunchenko, Anatoly P. Pushkarev

**Affiliations:** ^1^ Department of Optics and Nanophotonics Institute of Physics Kazan Federal University Kazan 420008 Russia; ^2^ School of Physics and Engineering ITMO University St. Petersburg 197101 Russia; ^3^ Center for Photonics and Quantum Materials Skolkovo Institute of Science and Technology 30/1 Bolshoy Boulevard Moscow 121205 Russia

**Keywords:** crystal‐liquid duality, disordered perovskite, electron‐photon interaction, electronic Raman scattering, near‐field photon momentum, photoluminescence blinking, Raman blinking

## Abstract

The interaction of light with solids can be dramatically enhanced owing to electron‐photon momentum matching. This mechanism manifests when light scattering from nanometer‐sized clusters including a specific case of self‐assembled nanostructures that form a long‐range translational order but local disorder (crystal‐liquid duality). In this paper, a new strategy based on both cases for the light‐matter‐interaction enhancement in a direct bandgap semiconductor – lead halide perovskite CsPbBr_3_ – by using electric pulse‐driven structural disorder, is addressed. The disordered state allows the generation of confined photons, and the formation of an electronic continuum of static/dynamic defect states across the forbidden gap (Urbach bridge). Both mechanisms underlie photon‐momentum‐enabled electronic Raman scattering (ERS) and single‐photon anti‐Stokes photoluminescence (PL) under sub‐band pump. PL/ERS blinking is discussed to be associated with thermal fluctuations of cross‐linked [PbBr_6_]^4‐^ octahedra. Time‐delayed synchronization of PL/ERS blinking causes enhanced spontaneous emission at room temperature. These findings indicate the role of photon momentum in enhanced light‐matter interactions in disordered and nanostructured solids.

## Graphical abstract

Light scattering from a perovskite semiconductor can be enhanced due to structural disorder leading to delocalization of optical near‐field and electron‐photon momentum matching. Electronic Raman scattering serves as an alternative mechanism for detrapping charge‐carriers from mid‐gap states into the conduction band. This mechanism explains Raman/photoluminescence blinking, near‐field photon reabsorption and the anti‐Stokes photoluminescence shift by 411 meV under sub‐band pump.

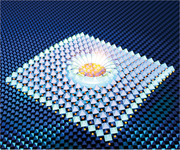

## 1 Introduction

Defects that inevitably occur in solids when natural growth or synthesis are mainly associated with static lattice imperfections (vacancies, impurities, interstitials, etc.) affecting the optical and electronic properties of crystals. In addition, thermal and polar fluctuations of a lattice can invoke dynamic disorder^[^ ^1^, ^2^ ^]^ that produces an electronic continuum of dynamic states across the forbidden gap, known as the Urbach bridge.^[^ ^3^ ^]^ Physically, it leads to the hybridization of static and dynamic defect states,^[^ ^4^, ^5^, ^6^ ^]^ allowing for charge carrier hopping either through inelastic light scattering or tunneling. This behavior is typical for solids exhibiting crystal‐liquid duality^[^ ^7^ ^]^ in which vibrations of coupled rigid frameworks are long‐range correlated. Such systems include perovskites,^[^ ^7^, ^8^, ^9^ ^]^ MXenes,^[^ ^10^, ^11^ ^]^ chalcogenides,^[^ ^12^ ^]^ liquid crystals,^[^ ^13^ ^]^ and high‐entropy crystals.^[^ ^14^ ^]^ Structural dynamics in their lattices can significantly influence the electronic polarizability, breaking the Franck‐Condon approximation.^[^ ^15^ ^]^ The static/dynamic states can be disentangled by using quantum confinement that increases a number of surface mid‐gap states^[^ ^16^ ^]^ and quenches temperature‐dependent structural fluctuations predominant in bulk crystals.^[^ ^1^ ^]^

A common feature of dynamic disorder is the onset of a wide Rayleigh wing^[^ ^17^ ^]^ or a low‐frequency Raman peak.^[^ ^1^, ^2^ ^]^ Nano‐crystals (NCs) flare a spectrally broad high‐energy emission that is red‐shifted and size‐dependent.^[^ ^18^ ^]^ In spatially‐confined metals, both emissions overlap completely and we observe a single band peaked at the pumping wavelength,^[^ ^19^ ^]^ whereas these are spectrally well‐resolved in disordered semiconductors.^[^ ^3^ ^]^ The high‐energy emission originates from inelastic light scattering by fluctuations of the electronic density in the vicinity of the Fermi level, as pioneered by A. Mal'shukov in 1989.^[^ ^20^ ^]^ To date, this effect is understood as electronic Raman scattering (ERS) in which initial and final electronic states are different, and optical transitions may be indirect due to the electron‐photon momentum matching.^[^ ^21^, ^22^, ^23^, ^24^ ^]^ This is achieved by generating a near‐field photon with increased momentum.^[^ ^25^ ^]^ The ERS is similar to the Compton effect^[^ ^26^ ^]^ for visible radiation in which near‐field photons are scattered by atomic‐scale fluctuations of the electronic polarizability in polar crystals.

Metal halide perovskite crystals possess a specific ABX_3_ structure in which the 4A and B cations interact with the halide 6X anions in such a way to form chemically stable 3D corner‐sharing octahedral rigid frameworks [BX_6_].^[^ ^4^, ^5^, ^6^, ^7^, ^8^, ^9^ ^]^ A direct bandgap semiconductor CsPbBr_3_ (*E_g_* = 2.37 eV) serves as a model system showing long‐range chains of cross‐linked [PbBr_6_]^4‐^ octahedra. Even though there are considerable advances in understanding the mechanisms of emission in perovskite crystals,^[^ ^27^, ^28^, ^29^, ^30^ ^]^ there is still a number of fundamental concerns that continue to drive interest of the scientific community. These include (1) highly‐emissive quantum dots in a glass matrix,^[^ ^31^, ^32^, ^33^ ^]^ (2) PL blinking,^[^ ^34^, ^35^, ^36^ ^]^ (3) enhanced PL when phase transitions,^[^ ^27^, ^37^ ^]^ (4) single‐photon anti‐Stokes photoluminescence (aS‐PL) under sub‐band pump,^[^ ^38^, ^39^, ^40^ ^]^ (5) PL redshift in quantum dots,^[^ ^18^, ^28^ ^]^ (6) super‐photoluminescence (super‐PL) in cross‐linked nanocrystals (or superlattices),^[^ ^41^, ^42^, ^43^ ^]^ (7) single‐photon super radiance in quantum dots,^[^ ^44^ ^]^ (8) low‐frequency Raman (disorder) peak^[^ ^1^, ^3^ ^]^ and (9) reversible crystal‐glass transition in perovskites.^[^ ^45^ ^]^

In this article, we develop a physical model based on ERS and address aforementioned challenges by using the concept of disorder‐order interface (DOI) which is a system of cross‐linked CsPbBr_3_ NCs and a host crystal.^[^ ^3^ ^]^ In this model, the NCs capture the light and generate photo‐electrons in the conduction band through the ERS, and then the charge carriers tunnel into the crystal and recombine radiatively. This is claimed by PL/ERS blinking caused by thermal fluctuations of cross‐linked [PbBr_6_]^4‐^ octahedra. In addition, PL/ERS kinetics recognizes enhanced spontaneous PL (ESPL) and spontaneous bunching PL as soon as PL/ERS blinking is synchronized. The ERS process is an alternative mechanism for detrapping charge carriers from mid‐gap states into the conduction band when phonon energy is insufficient.

## 2 Discussion and Results

**Figure 1:**
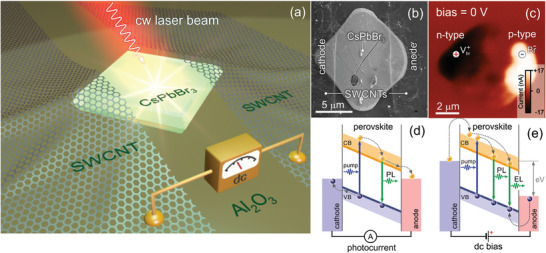
a) Artistic illustration of a CsPbBr_3_ pad, whose left and right corners are top‐covered with SWCNT electrodes, b) SEM image of the CsPbBr_3_ perovskite pad (top view). c) Photocurrent map across the perovskite pad under 633 nm cw illumination when dc bias off. d,e) Sketches of PL mechanisms when external dc voltage bias off and on.

Solution‐processed synthesis of perovskite crystals is a traditional route to engineer static defects that are still difficult to control.^[^ ^46^, ^47^ ^]^ Our strategy aims to generate static/dynamic defects using a pulsed dc bias applied to a pristine CsPbBr_3_ crystal pad, as‐grown on a sapphire substrate (see details in Experimental Section) as schematically shown in **Figure** **1**a. A PL study of CsPbBr_3_ at 473 nm indicates the band‐to‐band transition at ≈2.37 eV (523 nm) and confirms the spectral homogeneity across the crystal (Section I, Supporting Information). A detailed procedure for integrating the CsPbBr_3_ pad into the circuit in which electrodes are metallic‐type single‐walled carbon nanotubes (SWCNTs) can be found in refs. [48, 49]. Figure 1b shows a scanning electron microscopy (SEM) image of a metal‐semiconductor‐metal (MSM) structure enabling switchable photovoltaic effect (Figure 1c).^[^ ^50^, ^51^ ^]^ The photocurrent map exhibits negative (n‐type) and positive (p‐type) regions near cathode and anode, respectively. Photogenerated charge carries are separated by energy band bending due to Schottky contact, making the n‐i‐p junction in the MSM structure (Figure 1d). The change in dc polarity when raster scanning a 633 nm focused laser beam (no bias) is due to the mirror symmetry of the band bending at the opposite (n‐ and p‐type region) ends (Figure 1c).

Upon applying forward external dc bias, coupled ionic and electronic transport increases the concentration of bromine vacancies ($V_{\mathrm{Br}}^{+}$) and bromine anions (Br^−^) near the cathode and anode, respectively (Figure 1c).^[^ ^50^ ^]^ This creates a built‐in electrical field that balances and weakens the external electrical field. The internal electrical field modifies a current kinetics when circuit‐off noticeably, provided that the external bias was above 3 V (Figure S2a, Supporting Information).^[^ ^49^ ^]^ Sharp downward current peaks appear as a response to the rapid relaxation of electrons invoked by the built‐in electric field. This is precisely the voltage threshold at which a mixed ion‐electron semiconductor can serve as either an electroluminescent photodetector or a light‐emitting diode (LED).^[^ ^49^ ^]^ The dc current kinetics when circuit‐on indicates a charging process of the n‐i‐p junction. Sporadic current oscillations at 9 V stem from light‐enhanced electroluminescence (EL) (Figure 1e; Figure S2b, Supporting Information) that blink, as seen in Movie 1. The EL occurs solely at the n‐type region (Figure 1c; Figure S3, Supporting Information). The observed asymmetry is caused by intrinsic imperfections of a perovskite crystal and non‐uniform SWCNT electrodes, which unbalance the distribution of charge carriers, resulting in asymmetric energy bending at opposite SWCNT electrodes. Figure S3a,d (Supporting Information) shows the sixfold increase in the EL intensity at the n‐type region under direct bias compared to that at the p‐type region under reverse bias. The EL signal disappears for both regions when the voltage polarity is inverted (Figure S3b,c, Supporting Information). In the n‐type region, the fast thermalization of hot electrons generates enough heat enabling to modify the sample chemically and structurally in those areas that shined (Figure S4a–d, Supporting Information). It is important to highlight that a green PL photon stemming from interband radiative recombination is spatially confined and, therefore, it has the expanded momentum, allowing indirect optical transitions to populate the conduction band excessively. In addition, one should mention current‐induced Joule heating that competes with optical heating owing to parasitic re‐absorption of green emission. In general, these processes hold simultaneously and their separation is not a trivial task.^[^ ^52^, ^53^ ^]^

The multi‐pulsed dc bias locally creates a disordered state that imbalances the primary distribution of charge carriers. This state resembles glass^[^ ^54^ ^]^ consisting of randomly oriented nanoclusters with a composition close to such perovskite phases as CsPbBr_3_, CsPb_2_Br_5_, and Cs_4_PbBr_6_. Local optical heating of a CsPbBr_3_ crystal leads to its structural deformation violating the long‐range translational order. In addition, ERS‐induced optical heating invokes chemical transformation of the nanoclusters and, as a result, a mixture of disordered quasi‐perovskite nanoclusters is formed.

**Figure 2:**
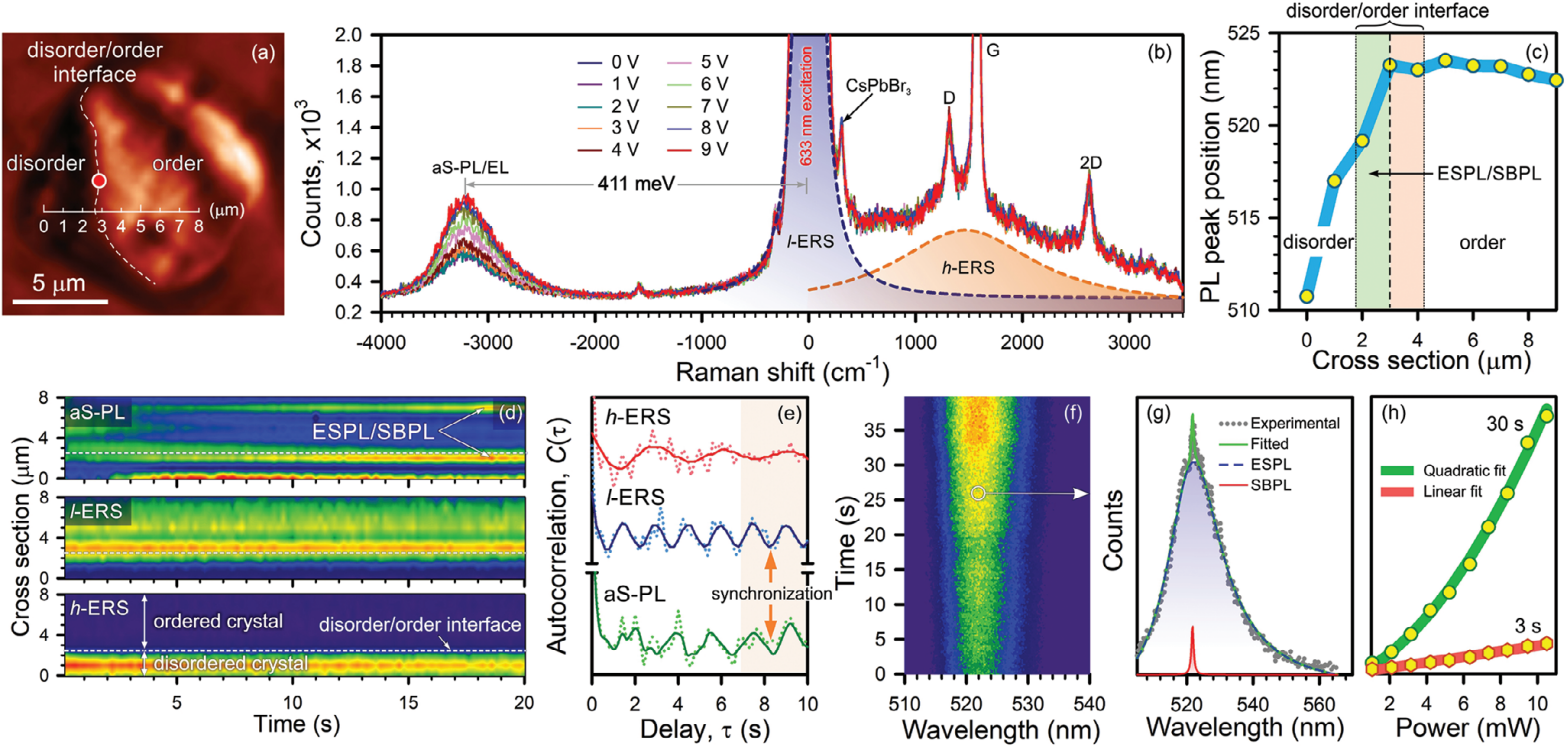
a) A confocal optical image of a CsPbBr_3_ pad consisting of disordered and ordered regions separated with the DOI. b) Raman spectra of a CsPbBr_3_ pad exposed to 633 nm excitation pump with the intensity of 0.4 MW cm⁻^2^ and different dc bias, registered at the DOI (red spot in Figure 2a). c) A plot of PL peak position versus cross section shown in Figure 2a. d) aS‐PL, *l*‐ERS and *h*‐ERS kinetics along a cross section marked in Figure 2a. e) Autocorrelation function for three processes registered at the DOI. f) 1D Raman map versus time. g) A PL spectrum at the spot marked in Figure 2f and its numerical decomposition into the ESPL (dashed blue curve) and spontaneous bunching PL (SBPL) (solid red curve) bands. h) Pump‐dependent PL intensity at the DOI after 3 s and 30 s.

Destructuring stops when charging flips into discharging, resulting in downward current kinetics (Figure S4e, Supporting Information). In this area, micro‐sized CsPbBr_3_ domains can transform into Cs_4_PbBr_6_ (*E_g_* = 3.9 eV) NCs at temperatures above 350 °C,^[^ ^55^ ^]^ which are easily recognized by low‐frequency Raman spectroscopy. Figure S5 shows (Supporting Information) Raman spectra of as‐synthesized pristine CsPbBr_3_ and Cs_4_PbBr_6_ for further analysis. Intense heating of shining areas of the perovskite crystal is supported by its low thermal conductivity (0.5 W m^−1^K^−1^),^[^ ^56^ ^]^ making it difficult to scatter heat. As mentioned above, the temperature increase during EL emission is achieved by optical transitions from shallow/deep traps into the conduction band, followed by electron thermalization and heat release (Figure S4c,d, Supporting Information). The structurally modified and intact regions are separated by a 2D DOI (**Figure** **2**a), consisting of long‐range coupled crystalline and nano‐crystalline structures.

Figure 2b shows dc‐induced Raman spectra of CsPbBr_3_ registered at the DOI (red spot in Figure 2a) exposed to a sub‐band pump (632.8 nm, 1.959 eV) with the intensity of 0.4 MW cm⁻^2^ for 10 s. Along with vibrational modes attributed to pristine CsPbBr_3_ at 310 cm^−1^ (2LO Pb‐Br stretching mode)^[^ ^57^ ^]^ and metallic SWCNTs at 1360 cm^−1^ (D‐mode), 1590 cm^−1^ (G‐mode) and 2720 cm^−1^ (2D‐mode),^[^ ^58^ ^]^ we can observe three broad emissions: (a) single‐photon anti‐Stokes PL/EL, (b) low‐energy electronic Raman scattering (*l*‐ERS) and (c) high‐energy electronic Raman scattering (*h*‐ERS).^[^ ^3^ ^]^ Both *l*‐ERS and *h*‐ERS, reconstructed with a regularized least‐square method, originate from the electron‐photon momentum matching owing to the increased momentum of a near‐field photon generated by quantum confinement and long‐range structural fluctuations, respectively. This is the result of the non‐propagating optical near‐field possessing at least one imaginary wavenumber component.^[^ ^59^ ^]^

Overall, quantum confinement and long‐range fluctuations can be characterized by a correlation length *l_c_* that is similar to excitonic delocalization extent.^[^ ^44^ ^]^ In the first case, this is precisely the NC size, the latter determines a spatial extend for in‐phase oscillations of rigid frameworks. When *l_c_* ≪ λ, this effect can be understood in terms of the dipole‐dipole interaction between electronic and off‐center ion polarizability.^[^ ^2^ ^]^ This formalism explains the origin of a broad Rayleigh wing in highly associated liquids.^[^ ^17^, ^60^ ^]^ However, it is insufficient for a system possessing crystal‐liquid duality^[^ ^7^ ^]^ in which the correlation length can increase dramatically (*l_c_* ≤ λ). In this case, inelastic low‐frequency scattering is associated with long‐range structural oscillations rather than rotation and translation of coupled light molecules, as previously thought.^[^ ^60^ ^]^ This means that a central (disorder) peak observed in such systems is specifically the Raman scattering^[^ ^1^, ^2^ ^]^ or *l*‐ERS^3^ (Figure S6, Supporting Information).

The central Raman peak is driven by the spontaneous long‐range structural correlations that are sensitive to temperature. Upon cooling down to 80 K, this peak disappears completely for CsPbBr_3_.^[^ ^1^ ^]^ In addition, temperature‐dependent phase transitions^[^ ^27^ ^]^ can affect the dynamic disorder in CsPbBr_3_ crystals. Figure S7 (Supporting Information) shows a temperature dependence of the Raman peak linewidth, termed as Urbach energy,^[^ ^3^ ^]^ when heating/cooling within the range of 20 ÷ 160 °C. It is important to note that the Urbach energy exhibits an anomalous rise in the tetragonal β‐phase when cooling, resulting in enhanced PL at the phase transition β → γ.^[^ ^27^, ^37^ ^]^ This phenomenon, that is still poorly understood, could be linked to the formation of longer structural chains through relaxing internal stresses at the α → β transition (Figure S7, Supporting Information).

Current‐induced order‐to‐disorder transformation occurs in regions that emit the EL, as shown in Figure S8a (Supporting Information). The *l*‐ERS intensity is independent of dc bias when EL is off and increases otherwise. In the disordered region, the EL intensity decreases due to the chemical transformation of CsPbBr_3_ to Cs_4_PbBr_6_ and the formation of NCs, suppressing the *l*‐ERS intensity (Figure S8b, Supporting Information). This means that quantum confinement prevents long‐range structural correlations that allow inelastic scattering of incident light. On the other hand, NCs generate near‐field photons with large momenta in magnitude, which allow optical transitions from mid‐gap states to the conduction band, resulting in *h*‐ERS (Figure S9, Supporting Information). The heavy *h*‐ERS tail extending beyond 3000 cm^−1^ (>0.4 eV)^[^ ^3^ ^]^ indicates the formation of tiny NCs with a huge density of surface mid‐gap states. In contrast to *l*‐ERS, the *h*‐ERS band is red‐shifted and its intensity increases noticeably when moving from order to disorder (Figure S9, Supporting Information).

The electron‐photon momentum matching governs both the processes, *l*‐ERS and *h*‐ERS, through long‐range structural fluctuations in crystals and quantum confinement in NCs, respectively. The vibrational Raman intensity defined as $I_{nm}∼\left\langle {\overset{↔}{\alpha }}_{nn}^{*}{\overset{↔}{\alpha }}_{mm}\right\rangle$ (where $\overset{↔}{\alpha }$ is an atomic polarizability tensor for two quantum states ψ_*n*_ and ψ_*m*_, 〈⋅⋅⋅〉 is an averaging symbol) can be modified for the ERS intensity, which is a function of correlation length *l_c_* or wavenumber *k*_0_, as follows
(1)

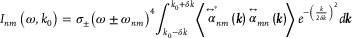

where σ_±_ are the cross‐sections for anti‐Stokes and Stokes scattering, the atomic polarizability taking into account the phase of incident radiation when r∼λ has the following view:

(2)

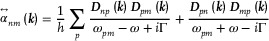

where ***D***_*nm*_ (***k***) = 〈ψ_*n*_|*e* ^***k*** (*r*) ***r***^∂/∂ ***r*** |ψ_*m*_〉 is the transient electrical dipole moment between electronic states ψ_*n*_ and ψ_*m*_, Γ is the width of electronic level. The momentum of a confined photon fluctuates around *k*_0_ ≅ π/*l_c_* within δ*k*.^[^ ^22^, ^23^ ^]^ The uncertainty δ*k* follows from the Heisenberg principle due to quantum confinement. For this reason, 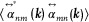 is averaged using the Gaussian momentum distribution in *k*‐space. In the long‐wavelength approximation *r* ≪ λ, a quantum system can be seen as a set of quantum oscillators with electrical dipole moments $D_{nm\;}e^{i\omega_{nm}t}$, in other words, the phase of light wave is constant for all oscillators. Otherwise, it is necessary to take into account the phase allowing to perceive the quantum system as currents and charges distributed in space.

Unlike the *l*‐ERS and *h*‐ERS bands which are not sensitive to applied external voltage (Figure 2b), the aS‐PL/EL intensity increases at the DOI exposed to both dc bias and sub‐band pump. A single‐photon up‐conversion follows from the linear behavior of the pump‐dependent PL intensity as shown in Figure S10 (Supporting Information). Despite partial destruction, the crystal retains its luminescent properties, but the PL blueshift indicates the presence of NCs at the disordered region (Figure 2c).^[^ ^61^ ^]^ This plot clearly visualizes the DOI of 2 µm in width (axial size) along the scale bar depicted in Figure 2a. aS‐PL, *l*‐ERS and *h*‐ERS kinetics along the scale bar shed light on the mechanisms of emission from cross‐linked crystalline and nano‐crystalline structures at the DOI (Figure 2d). The kinetics maps were built up with the temporal and spatial resolution of 200 ms and 500 nm, respectively. Through the entire crystal, the aS‐PL intensity drops in time, except the DOI and a region at 7 µm, in which it starts to grow up in 6–8 s. The fact that the *l*‐ERS intensity is time‐independent in crystal, disappears in the disordered region and increases at the DOI confirms the above hypothesis about long‐range structural fluctuations. Yet, this concept is supported by the absence of *h*‐ERS in crystal and the downward kinetics in the disordered region. A calculation of autocorrelation function, C(τ)=∫I(t)I(t−τ)dt, claims that all three emissions blink with the characteristic period: aS‐PL – 1.9 s, *l*‐ERS – 1.5 s and *h*‐ERS – 3 s (Figure 2e). A close inspection of the autocorrelation for aS‐PL indicates two periods: 1.5 and 1.9 s. Importantly, there is no a specific PL blinking period typically linked to the detection threshold.^[^ ^35^ ^]^ The increase in the aS‐PL intensity over time is associated with its reabsorption, saturating the crystal conduction band with free charge carriers (Figure S11, Supporting Information). The periodic oscillations of the *l*‐ERS intensity in the crystal indicate thermal fluctuations of a cross‐linked [PbBr_6_]^4‐^ octahedra network (Figure 2e). The decay kinetics of the *h*‐ERS intensity results from the depleted population of charge carriers residing the surface mid‐gap states in NCs. Once this process terminates both the aS‐PL and the *l*‐ERS begin to synchronize with the period of 1.5 s, and we observe ESPL and sporadic narrow peaks (spontaneous bunching PL (SBPL)) simultaneously, as follows from 1D aS‐PL kinetics (Figure 2f; Figure S11, Supporting Information). The SBPL process closely resembles amplified spontaneous emission,^[^ ^62^ ^]^ an important phenomenon for mirrorless lasing at room temperature.^[^ ^63^ ^]^

The ESPL is driven by the free‐carrier recombination by which all emitted photons are incoherent. The narrow peaks, randomly distributed over the ESPL band (Figure 2f), appear due to the avalanche optical transitions of bunched charge carriers emitting coherent photons. However, the photons emitted by different bunches are incoherent. This bunching effect can be explained by a large polaron formation^[^ ^7^ ^]^ in the crystal surrounded by a huge number of NCs being a charge‐carrier reservoir (Figure S12, Supporting Information). This DOI might likely enable true random lasing^[^ ^64^ ^]^ when cooling below 100 K.^[^ ^63^ ^]^

The aS‐PL band, observed in our experiment, is decomposed into the elementary peaks, the broad ASE band (the product of the Lorentzian profile and the exponential function taking into account the Urbach tail) and the Lorentzian‐shaped narrow BSE band, using a regularized least‐square method (Figure 2g). The pump‐dependent aS‐PL intensity measured in 3 and 30 s exhibits linear and quadratic behaviors. The latter excludes two‐photon absorption (Figure S10, Supporting Information), and indicates the nonlinear regime at the DOI with the temporal threshold of 6–8 s (Figure 2h). Initially, the excitonic radiative recombination is predominant owing to the lower concentration of free charge carriers in the crystal exposed to the sub‐band pump. Once the re‐absorbed green PL saturates the conduction band of the crystal with free charge carriers, the radiative recombination exhibits the electron‐hole (quadratic) behavior (Figure 2h; Figure S10, Supporting Information).^[^ ^65^, ^66^ ^]^ These effects are basically played in the disordered region within the transition zone, as marked in Figure 2c. Time‐delayed synchronization of the aS‐PL and *l*‐ERS blinks (Figure 2e) is achieved by using the sporadic green PL as a feedback. Unlike conventional perovskite crystals, the DOI contains an inexhaustible reservoir of trapped charge carriers in NCs, which are detrapped by using the *l*‐ERS mechanism and moved into the crystal conduction band (Figure S11, Supporting Information).

A total blinking period τ of aS‐PL at the DOI is determined by the sum of the charge‐carrier lifetime τ_*NC*_ within the NC bandgap, the nanocrystal‐to‐crystal transition (tunneling) time τ_*NC* → *C*_ and the lifetime of an electronic level in the conduction band edge τ_*C*_: τ = τ_*NC*_ + τ_*NC* → *C*_ + τ_*C*_. Since τ_*NC*_ ≫ τ_*NC* → *C*_ > τ_*C*_, it is sufficient to estimate τ_*NC*_ per unit volume when an electron resides the Urbach bridge:

(3) $$\tau_{NC}≅E_{g}\frac{dn}{dE}N\tau_{0}$$

where *N* is the concentration of static defects (estimated to be 10^15^÷10^16^ cm^−3^),^[^ ^35^ ^]^ *E_g_* is the bandgap energy (2.37 eV for CsPbBr_3_), τ_0_ is a period of thermal fluctuations of [PbBr_6_]^4‐^ octahedra, generally varying between 1 ps and 10 ps,^[^ ^40^, ^67^ ^]^ and *dn*/*dE* is the density of dynamic states across the Urbach bridge, defined as

(4) $$\frac{dn}{dE}∼\frac{1}{e}{\left(\frac{l_{c}}{\lambda }\right)}^{d}$$

here *e* is the charge of an electron, λ is the pumping wavelength (632.8 nm), *d* is the topological dimension (*d* = 3 for bulk crystal). The correlation length *l_c_* changes from 1 nm to 100 nm (the upper limit is driven by the lateral size of twin domains, measured with tip‐enhanced Raman scattering microscopy (Figure S13, Supporting Information), the blinking period varies between microseconds and tens of seconds, which is consistent with earlier theoretical predictions.^[^ ^35^ ^]^ This characteristic time can monitor with the naked eye within a few seconds, as seen in Movie 1 – the EL sporadically emits when applied dc bias of 9 V.

**Figure 3:**
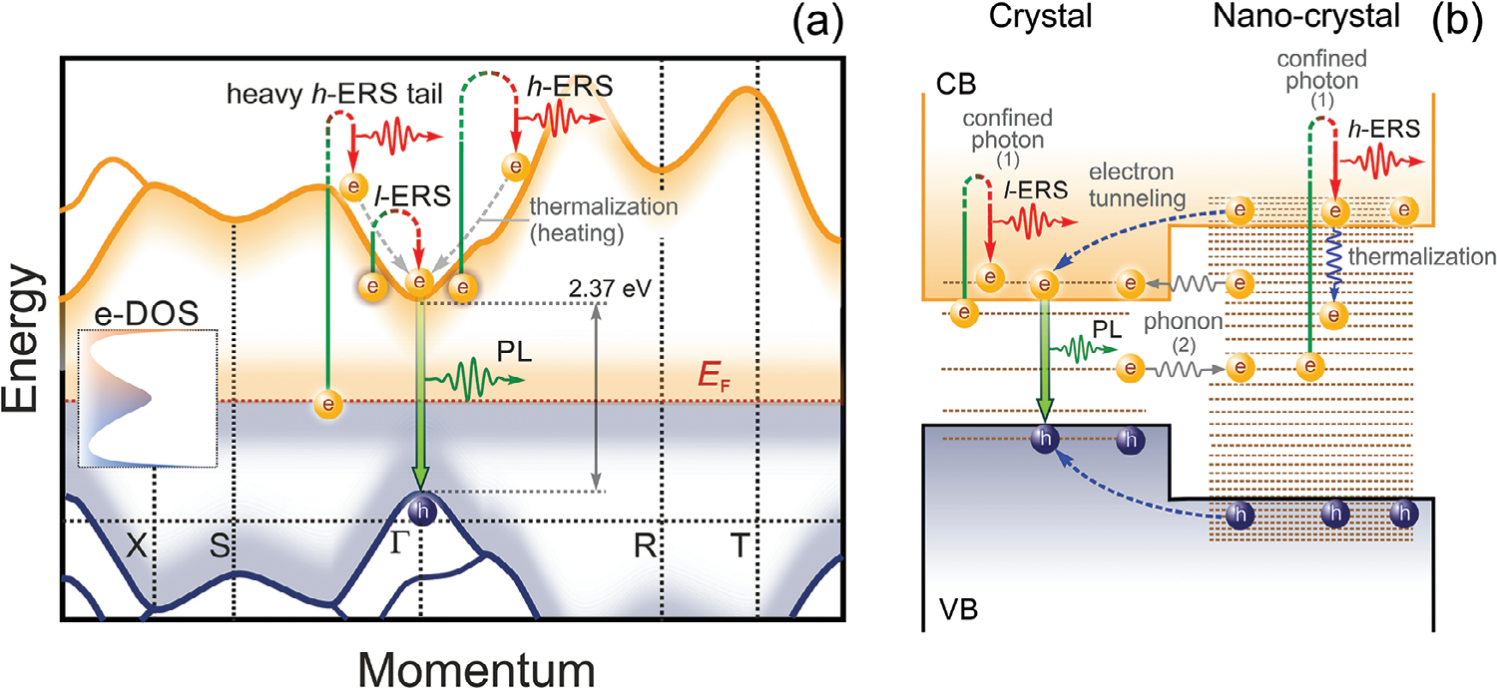
a) Energy band structure of CsPbBr_3_. b) Schematic illustration of optical transitions at the DOI (not to scale).

Using the ERS concept, we suggest an alternative mechanism of up‐conversion photoluminescence (PL) in metal halide perovskites under sub‐band pump.^[^ ^38^, ^47^, ^68^, ^69^, ^70^ ^]^ One of the fundamental barriers to explain anti‐Stokes shifts of a few hundred meV is insufficient energy of phonons to move charge carriers from the deep states to the conduction band. Bo Wu et al. interpreted this effect using thermal fluctuations of coupled rigid frameworks making the bandgap to oscillate.^[^ ^40^ ^]^ As a result, the charge carriers can tunnel from traps to the conduction band. In our model, shown in **Figure** **3**a, the anti‐Stokes shift by 411 meV (Figure 2b) can be interpreted through the *l*‐ERS in crystal and the *h*‐ERS in NC, in which the momentum of an incident photon is expanded by either long‐range structural oscillations of [PbBr_6_]^4‐^ octahedra or quantum confinement, respectively. It is important to stress that the *l*‐ERS process enables to drag trapped electrons from shallow states into the conduction band predominantly, whereas the *h*‐ERS process moves the electrons from all traps within the forbidden gap. Those charge carriers that transfer from the mid‐gap states into the conduction band contribute to the heavy *h*‐ERS tail (Figure 3a; Figure S9, Supporting Information).

Let us consider a model system of coupled crystal and NC structures to explain the enhancement of the emissions at the DOI upon sub‐band pump (Figure 3b). There are two mechanisms making the trapped electrons to travel between the localized and extended states: (1) electron‐photon interaction^[^ ^3^ ^]^ and (2) electron‐phonon interaction.^[^ ^71^, ^72^ ^]^ While phonon‐assisted indirect optical transitions are insufficient to observe inelastic broadband emissions, a concept of confined photon with increased momentum can shed light on the origin of observable emissions. Sub‐wavelength light localization in crystal, driven by long‐range structural fluctuations, increases photon momentum insignificantly. This leads to temperature‐dependent *l*‐ERS throughout the crystal, but this process disappears at the disordered region due to quantum confinement (Figure 2d). This is precisely the emission mechanism that is responsible for PL blinking. It is important to stress that the density of dynamic states in a perovskite crystal exceeds that of static states. The picture is opposite in NCs wherein the density of surface static states is maximal. The highly confined photon initiates indirect optical transitions from the mid‐gap states to the conduction band, which predominantly occur at the disordered region and emit the *h*‐ERS (Figure 2d). This emission is red‐shifted by a few hundred meV, and previously interpreted as PL.^[^ ^16^ ^]^ An electron in the NC conduction band is either thermalized back to populate a static defect state or tunnel into the crystal conduction band for radiative recombination. The green PL is subsequently reused for band‐to‐band pumping.^[^ ^73^ ^]^ This mechanism allows a PL broadening and blueshift near the DOI (Figure S14, Supporting Information). The enhanced *l*‐ERS process (Figure 2d) can greatly pump the conduction band using confined photons, further followed by excess radiative recombination in the band‐to‐band region (Figure S14e, Supporting Information). In this figure, we see the inhomogeneous broadening of the PL band caused by the larger cross‐section of band‐to‐band absorption in contrast to that of the sub‐band absorption near the DOI. Photon‐momentum‐enabled interband optical transitions drive the PL blueshift that was previously perceived as PL of quantum dots formed in the crystal (Figure S14e, Supporting Information).^[^ ^32^, ^33^ ^]^

**Figure 4:**
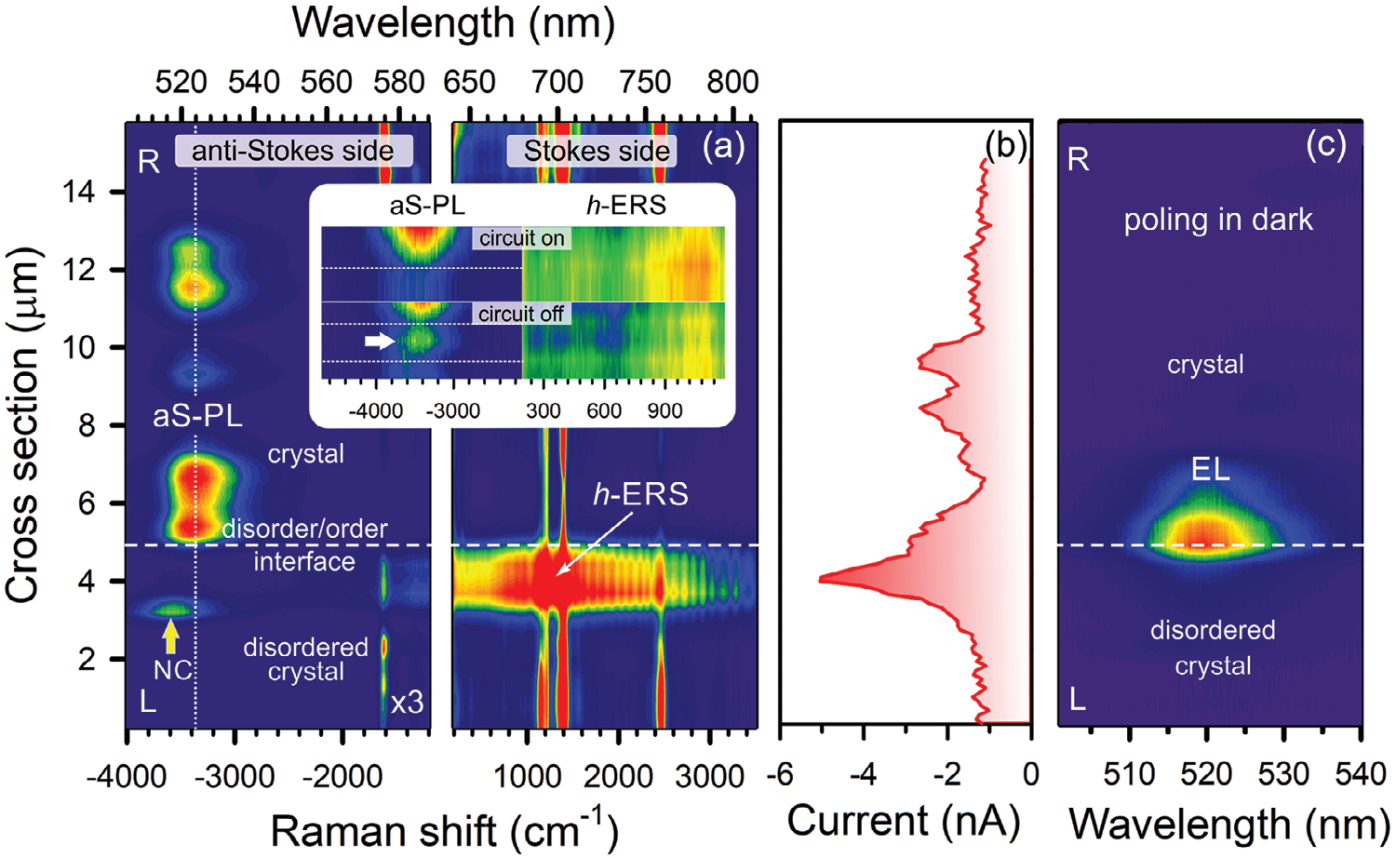
a) 1D Raman intensity map, b) a photocurrent distribution curve and c) 1D EL map at dc bias of 7 V along the cross section highlighted in Figure 2a. The inset in the left panel shows the aS‐PL and *h*‐ERS at the DOI when the circuit is on and off. The dotted straight lines mark the *h*‐ERS maximum. The arrow indicates an additional aS‐PL flare while circuit off.

**Figure** **4**a shows a 1D Raman intensity map across the CsPbBr_3_ crystal pad exposed to sub‐band pump at 633 nm. This map clearly confirms the physical model in Figure 3b. The *h*‐ERS and aS‐PL are enhanced near the DOI on opposite sides, respectively. The intensity of the anti‐Stokes side was increased by a factor of 3 for easy comparison of the anti‐Stokes and Stokes signals in Figure 4a. In the disordered region, we find a NC emitting the PL at 516 nm as marked with the arrow. The enhancement of the *h*‐ERS is accompanied by an increase in current (Figure 4b), which indicates an excess of photo‐generated electrons in the conduction band. Upon moving from disorder to order, the current drops due to the tunneling of photo‐generated electrons into the crystal wherein they recombine to emit the aS‐PL. This observation holds the above hypothesis that the *h*‐ERS process moves charge carriers from traps within the bandgap into the conduction band (Figure 3b). While the circuit is on, the *h*‐ERS signal is maximum and the aS‐PL is minimum at the DOI as seen from the inset in Figure 4a. Once the circuit is turned off, excess photo‐generated free charge carriers begin to tunnel into the crystal, and we observe an extra aS‐PL flare, marked with the arrow in the inset (Figure 4a). This is a strong argument in favor of enrichment of the conduction band with free charge carries transferred from the Urbach bridge using the *h*‐ERS process. In contrast, the *l*‐ERS process, not shown in Figure 4a, is insensitive to circuit switching (Figure S15, Supporting Information) since most charge carriers remain to be trapped within the bandgap when soft inelastic light scattering (Figure 3b).

Figure 4с shows the EL generation near the DOI in the dark when external applied voltage of 7 V. Unlike the initial crystal that sporadically emits the EL in the n‐type region, the formation of a DOI allows the n‐i‐p junction to be tuned for radiative recombination.

Thus, this result can be used for producing 3D ordered bulk systems to improve emission efficiency, e.g., as seen from recently synthesized multicomponent nanocrystal superlattices.^[^ ^74^ ^]^ Reversible disorder‐to‐order transitions^[^ ^45^ ^]^ in systems exhibiting crystal‐liquid duality^[^ ^7^ ^]^ may serve as a promising platform for neuromorphic computing.^[^ ^75^, ^76^ ^]^ The concept of expanded photon momentum allows not only explain the origin of observable inelastic broadband emissions, long observed in most different disordered systems, but also develop next‐generation optoelectronic technologies and devices.

## 3 Conclusion

In this work, we have developed a physical model based on ERS to address the enhanced emissions from a metal halide perovskite CsPbBr_3_, a semiconductor exhibiting crystal‐liquid duality. This model was successfully tested by using *a* DOI. The DOI on the CsPbBr_3_ pad is created through local heating caused by green EL. The underlying mechanism is related to near‐field photons possessing expanded momenta, leading to the ERS. Due to the electron‐photon matching, the ERS process locally heats up a crystal significantly, making it disordered. The disordered crystal contains randomly oriented nanoclusters and nanocrystals with a composition close to such perovskite phases as CsPbBr_3_, CsPb_2_Br_5_, and Cs_4_PbBr_6_.

This physical model explains a mechanism of an anomalous anti‐Stokes shift by 411 meV for CsPbBr_3_ exposed to sub‐band pump. The incident far‐field photon is scattered by long‐range structural fluctuations of cross‐linked [PbBr_6_]^4‐^ octahedra or spatially confined NCs, and, as a result, its momentum expands. Finally, this leads to inelastic low‐ and high‐frequency ERS. The electron‐photon momentum matching allows optical transitions from shallow/deep states of the Urbach bridge into the conduction band, further followed by charge‐carrier tunneling from NCs to crystal for emitting up‐conversion PL under sub‐band pumping.

We have shown that PL/ERS blinking in a CsPbBr_3_ crystal is a signature of thermal oscillations of cross‐linked [PbBr_6_]^4‐^ frameworks that vanish upon cooling.^[^ ^35^ ^]^ The *h*‐ERS blinking decays rapidly enough (a few seconds) due to the depletion of electrons in the mid‐gap states, and then the synchronization of *l*‐ERS and aS‐PL occurs, resulting in both ESPL and spontaneous bunching PL. Electron transitions from mid‐gap states to the conduction band have been confirmed by the formation of the heavy *h*‐ERS tail (above 0.4 eV) upon sub‐band pump.

The results obtained in this study will be beneficial in diverse fields of optoelectronic and photovoltaic applications, including (a) white LEDs, (b) solar cells based on cross‐linked amorphous‐crystalline semiconductors, (c) Urbach‐energy‐based temperature sensors, and (d) structural analysis of defects in disordered solids. Last, our findings highlight the role of expanded photon momentum in inelastic broadband emission from nanostructured solids and media exhibiting crystal‐liquid duality.

## 4 Experimental Section

### Synthesis of a CsPbBr_3_ Pad

CsPbBr_3_ microplates on a sapphire substrate were synthesized by high‐temperature sublimation similar to the previously reported.^[^ ^49^ ^]^ First, the source glass substrate was prepared using solution‐processed synthesis. Then, the target sapphire substrate was cleaned consistently in water, isopropanol, and acetone for 5 min. After that, the two substrates were placed in the high‐temperature titanium hotplate (PZ 28‐3TD) with the 6 mm gap between them. The initial temperature was 250 °C. During the synthesis, the temperature first raised to 530 °C during 15 min, then, it held for 10 min, and, last, followed by lowering down to 485 °C during 10 min. As a result, CsPbBr_3_ microplates were sublimated on the sapphire substrate.

### Metal‐Semiconductor‐Metal Structure Fabrication

A single‐walled carbon nanotube (SWCNT) thin film (20 nm thickness) was dry‐transferred on top of the as‐grown CsPbBr_3_ microplates. To finish the device fabrication, a light conversion Pharos femtosecond laser (200 fs pulse duration, wavelength 1030 nm) was used to cut the SWCNT film into two pieces above the chosen perovskite microplate. In order to provide micrometer sized distance between the two SWCNT electrodes, the light was focused through a 50 × lens (NIR Mitutoyo, NA = 0.65) while the xy‐position was altered with a programable piezo controller (Standa). For the successful ablation, the laser fluence level was controlled to be below 0.1 J cm⁻^2^. For more details on the process of SWCNT ablation see references.^[^ ^48^, ^49^ ^]^ To perform electrical measurements, a silver paste was put on top of each of SWCNT electrodes as shown in Figure S16 (Supporting Information).

### Far‐ and Near‐Field Raman Spectroscopy and Microscopy

Raman spectra and maps were captured with a multi‐purpose analytical instrument NTEGRA SPECTRA™ (NT‐MDT) in upright configuration. The confocal spectrometer was wavelength calibrated with a crystalline silicon (100) wafer by registering the first‐order Raman band at 521 cm⁻^1^. A sensitivity of the spectrometer was as high as ≈1700 photon counts per 0.1 s provided that we used a 100 × objective (N.A. = 0.7), an exit slit (pinhole) of 100 µm and a linearly polarized light with the wavelength of 632.8 nm and the power at the sample of 10 mW. No signal amplification regimes of a Newton EMCCD camera (ANDOR) was used. Low‐frequency Raman measurements were performed using a  m Bragg notch filter (OptiGrate) with a spectral blocking window of 10 cm⁻^1^.

## Conflict of Interest

The authors declare no conflict of interest.

## Author Contributions

S.S.Kh. conceived the original ideas. E.I.B. and S.S.Kh observed ERS/anti‐Stokes PL effects and developed a physical model. A.A.M. and A.P.P. suggested the device design and analyzed its operating modes. I.A.M., A.A.M. and A.P.P. carried out perovskite growth and fabricated the device. A.G.N. synthesized single‐walled carbon nanotube thin films. S.S.Kh. supervised the whole project. The manuscript is mainly written by S.S.Kh. with the contributions of all authors.

## Supporting information

Supporting Information

Supporting Information

## Data Availability

The data that support the findings of this study are available in Supporting Information of this article.
