# Supplementary material for: Extreme Electron‐Photon Interaction in Disordered Perovskites
Source: Adv Sci (Weinh). 2024 Oct 2;12(5):2405709. doi: 10.1002/advs.202405709 (PMC11792048; doi:10.1002/advs.202405709)
Supplement: Supplementary file 1 — Supporting Information [file ADVS-12-2405709-s001.docx]

Extreme Electron-Photon Interaction in
Disordered Perovskites

*Sergey S. Kharintsev^1*^, Elina I. Battalova^1^, Ivan A. Matchenya ^2^, Albert G. Nasibulin^3^, Alexander A. Marunchenko^2^, and Anatoly P. Pushkarev^2^*

^1^Department of Optics and Nanophotonics, Institute of Physics, Kazan Federal University, Kazan 420008, Russia

^2^School of Physics and Engineering, ITMO University, St. Petersburg 197101, Russia

^3^Center for Photonics and Quantum Materials, Skolkovo Institute of Science and Technology, 30/1 Bolshoy Boulevard, Moscow 121205, Russia

skharint@gmail.com

**I. Photoluminescence study of a CsPbBr_3_ pad**


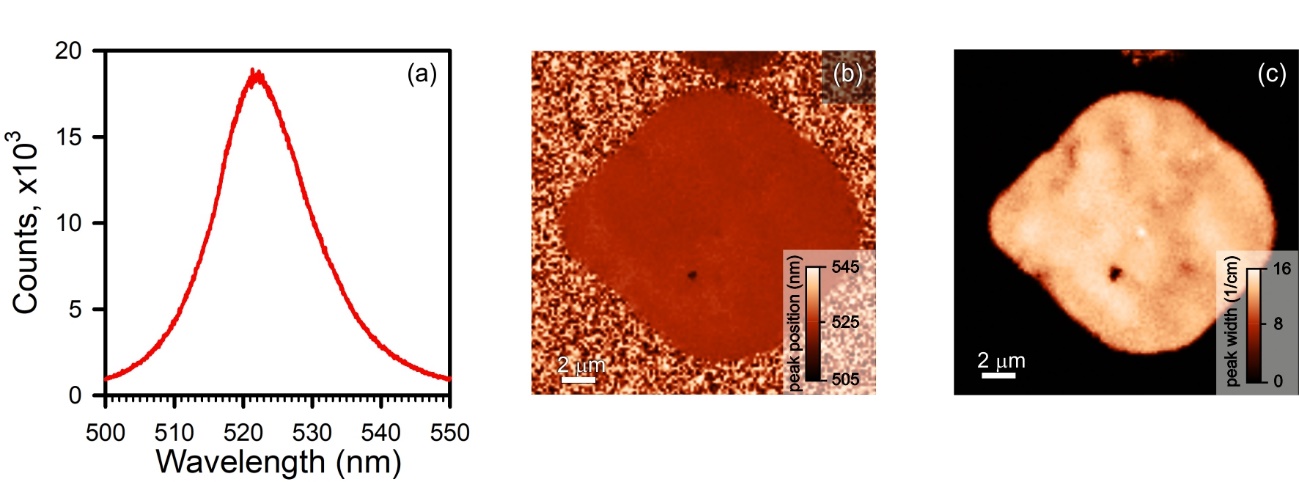


**Figure S1**. (a) PL spectrum at 473 nm excitation pump with the flux of 450 W/cm^2^ (exposure 0.1 s), (b) and (c) the peak position and linewidth maps (exposure 10 ms).

**II. Bias-assisted photoluminescence of CsPbBr_3_**


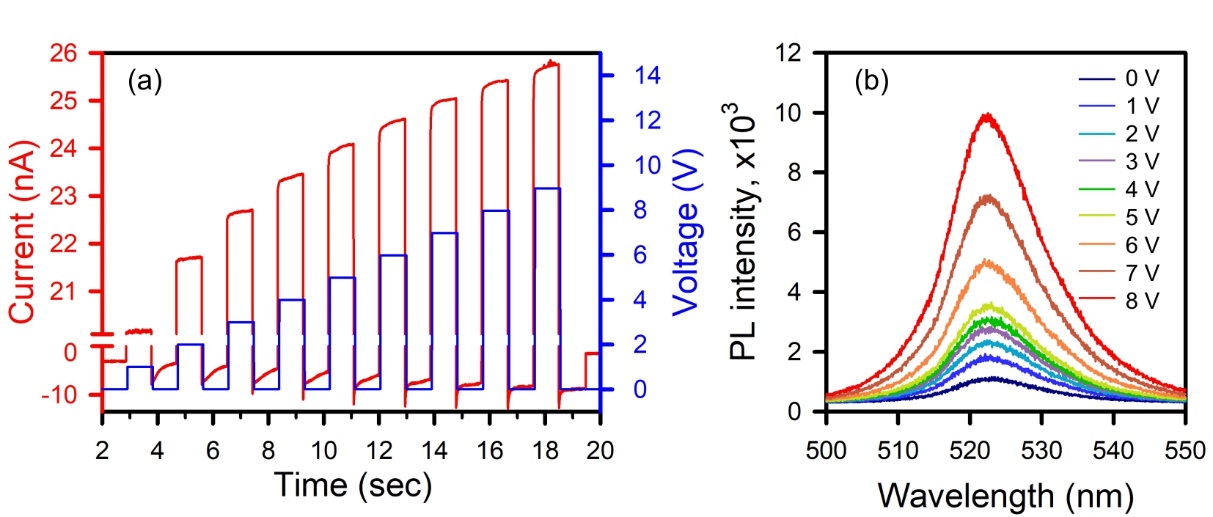


**Figure S2**. (a) Current and voltage kinetics of a CsPbBr_3_ pad under forward bias and 633 nm cw illumination, (b) bias-assisted PL of a CsPbBr_3_ pad exposed to 473 nm excitation pump with the flux of 180 W/cm^2^.

**III. Electroluminescence of CsPbBr_3_**


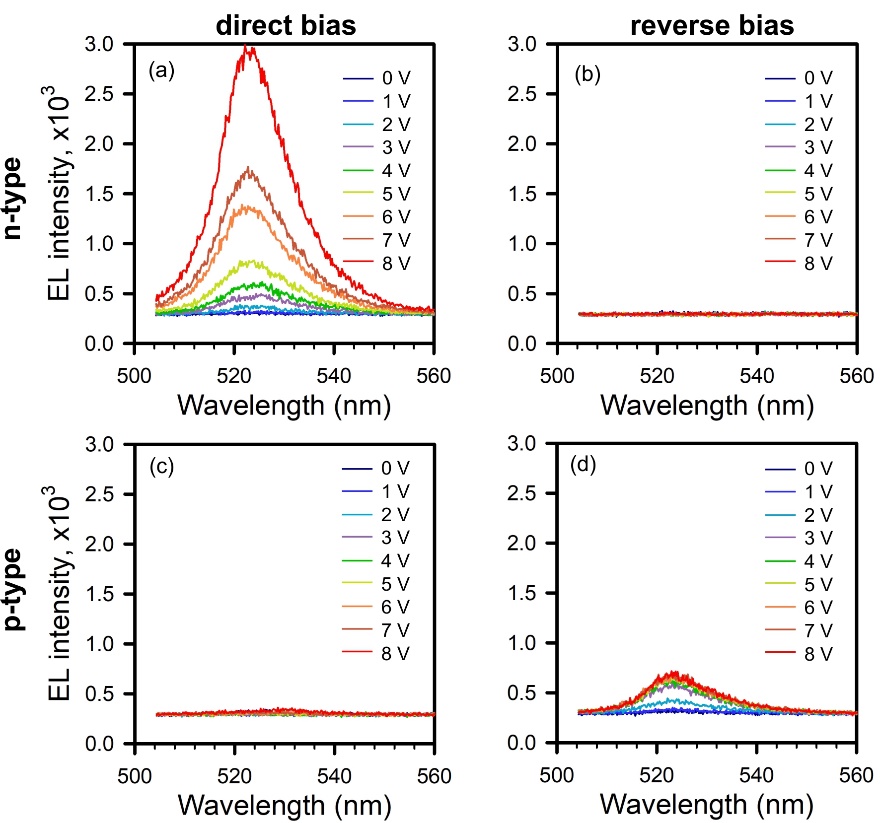


**Figure S3**. EL of a CsPbBr_3_ pad in dark at n-type and p-type regions under direct and reverse bias.

**IV. DC-induced disordering of a CsPbBr_3_ pad**


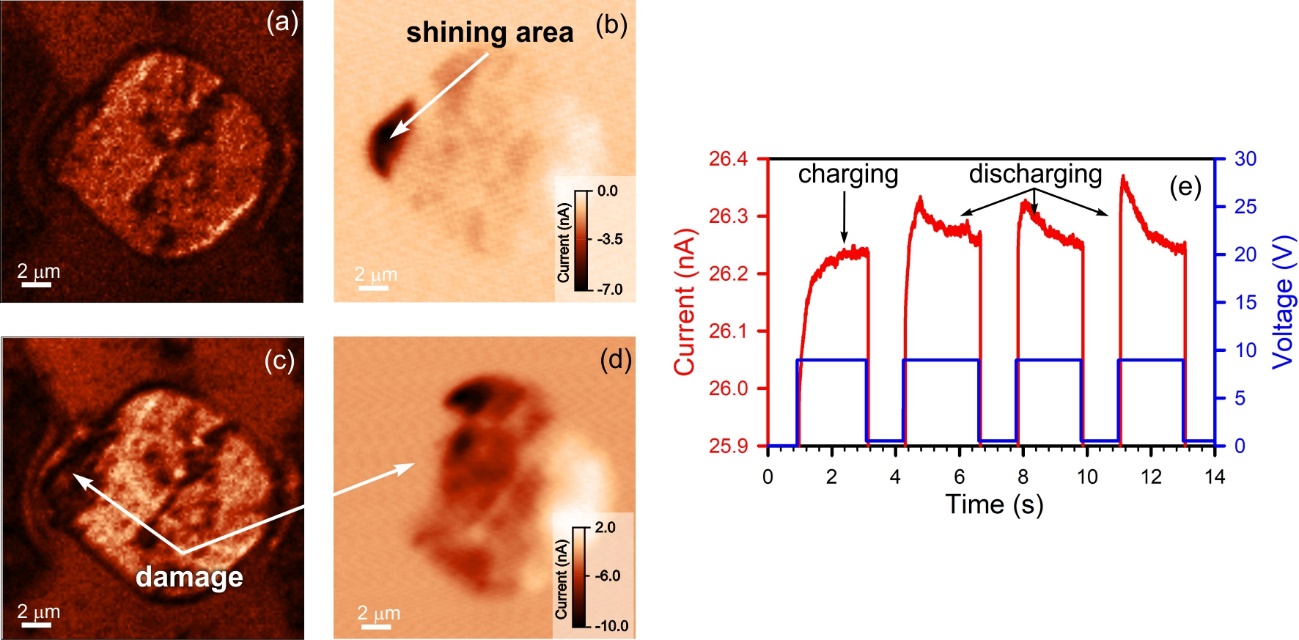


**Figure S4**. (a),(c) Confocal images of a CsPbBr_3_ pad and (b),(d) their dc current maps before and after damage of the left corner (shining area on movie 1). (e) Current kinetics under dc pulsed bias of 9 V (exposure 2 s).

**V. Low-frequency Raman spectroscopy of CsPbBr_3_ and Cs_4_PbBr_6_**


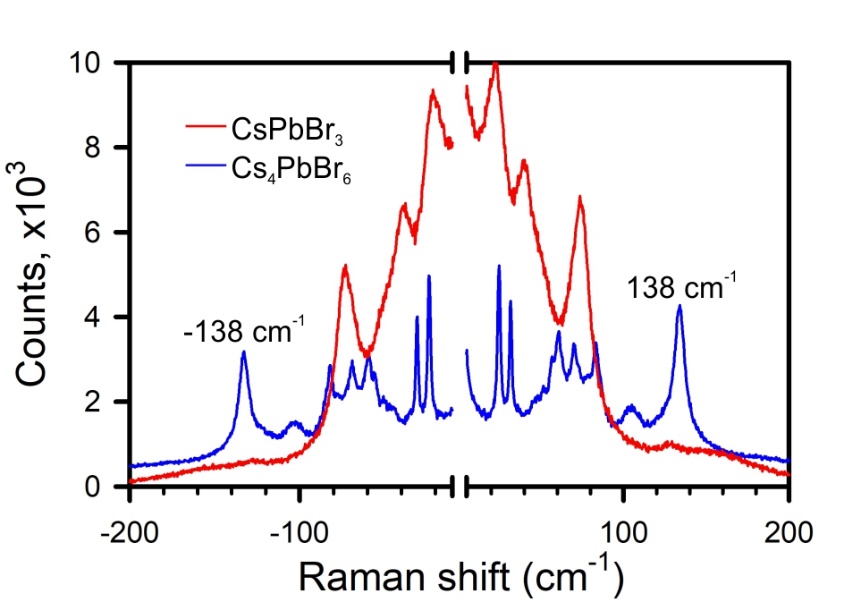


**Figure S5**. Low-frequency Raman spectra of CsPbBr_3_ and Cs_4_PbBr_6_.

**VI. A central (disorder) Raman peak in CsPbBr_3_**


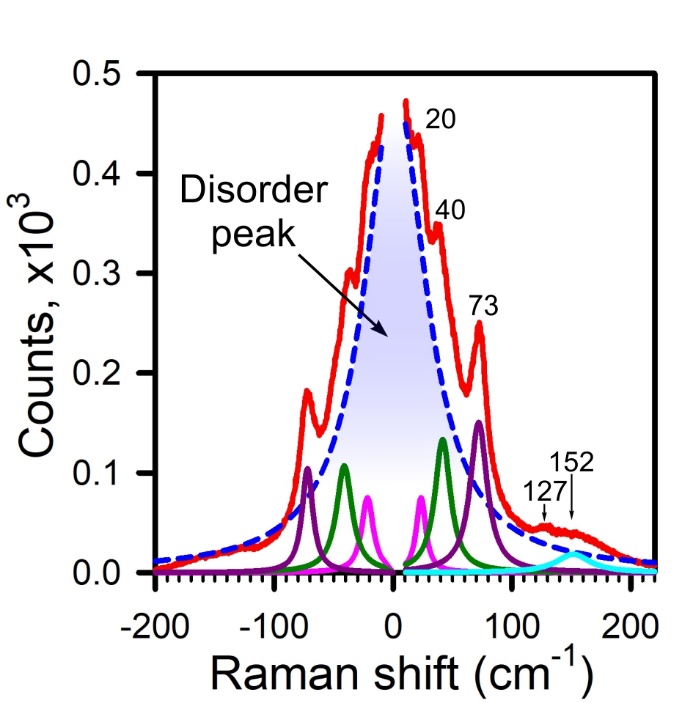


**Figure S6**. Low-frequency Raman spectrum of a CsPbBr_3_ pad under 633 nm excitation with the flux of 1 MW/cm^2^ (exposure 10 s) and its numerical decomposition into Lorentzians using a regularized least-square method.

**VII. Temperature-dependent Urbach energy**


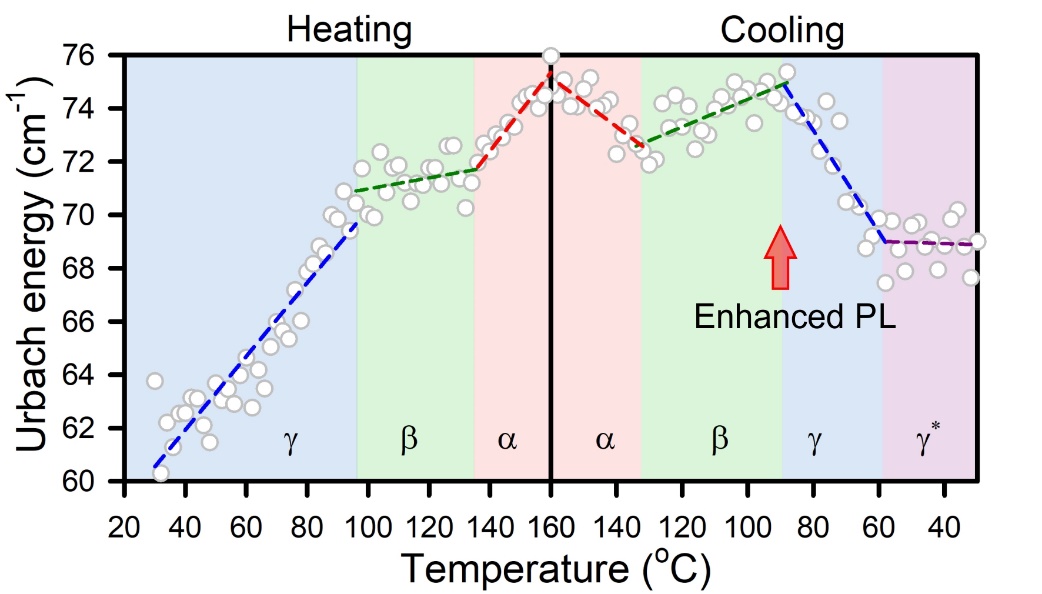


**Figure S7**. A plot of Urbach energy, defined as the full width at half height of *l*-ERS (a central Raman band (or a disorder peak) in Fig. S3 (a)), on temperature when heating and cooling of a CsPbBr_3_ pad with the rate of 2 K/s. Greek symbols stand for crystallographic phases of CsPbBr_3_: γ/γ* (orthorhombic), β (tetragonal) and α (cubic).

**VIII. Low-frequency Raman spectroscopy of ordered (crystalline) and disordered CsPbBr_3_ under dc bias**


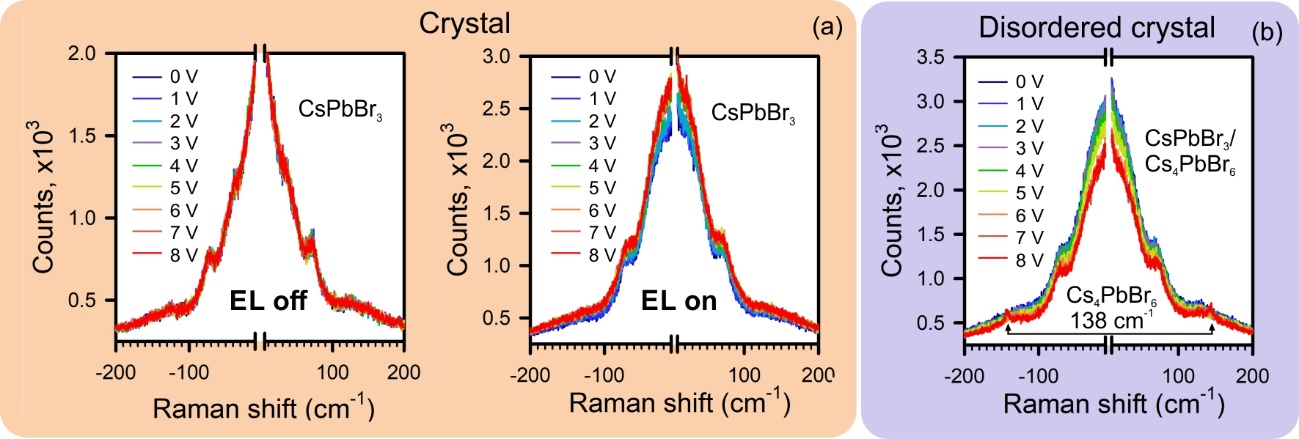


**Figure S8**. Low-frequency Raman spectra of a CsPbBr_3_ pad exposed to 633 nm excitation pump with the intensity of 0.4 MW/cm^2^ and different direct bias (exposure 1 s) in the ordered (crystalline) region (a) and disordered region (b).

**IX. High-energy electronic Raman scattering of a disordered/ordered CsPbBr_3_ pad**


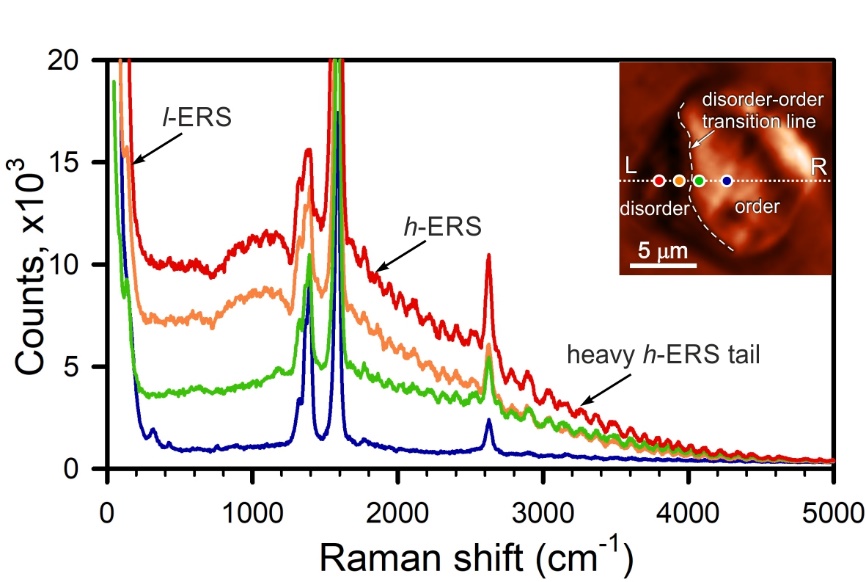


**Figure S9**. High-energy electronic Raman spectra of a CsPbBr_3_ pad captured at colorful spots (exposure 10 s), shown in the inset, exposed to 633 nm excitation pump with the fluence of 0.4 MW/cm^2^ (no bias).

**X. Pump-dependent Raman spectroscopy of a CsPbBr_3_ pad: the case of sub-band pumping**


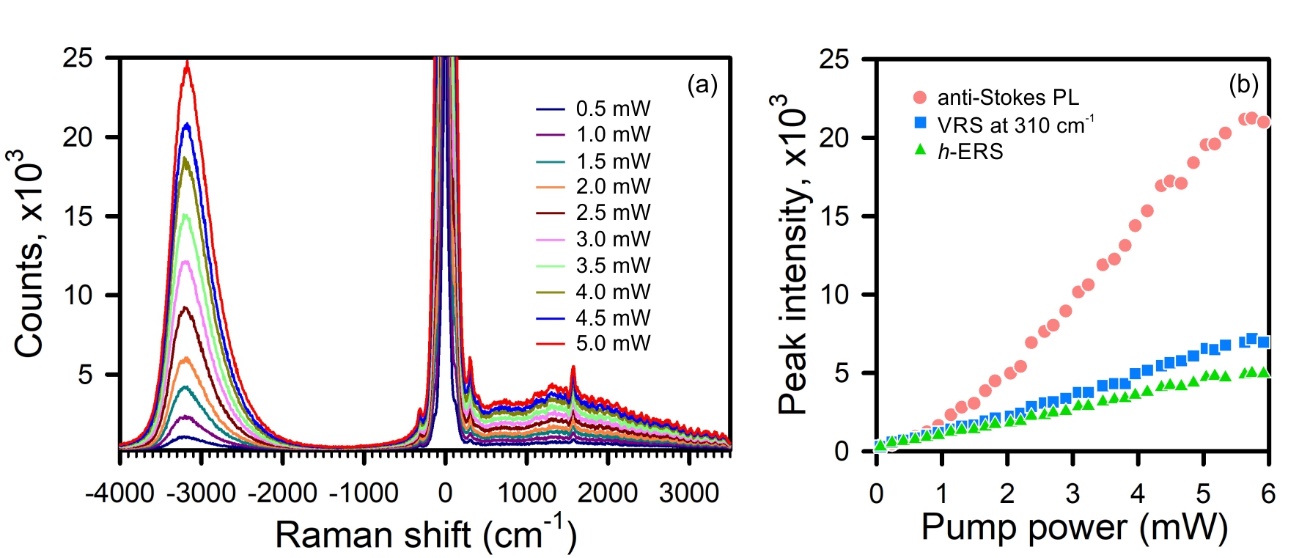


**Figure S10**. (a) Raman spectra of a CsPbBr_3_ pad while different pump, (b) a plot of the peak intensity vs the pump power for anti-Stokes PL, VRS at 310 cm^-1^ and *h*-ERS.

**XI. Enhanced spontaneous PL and spontaneous bunching PL**


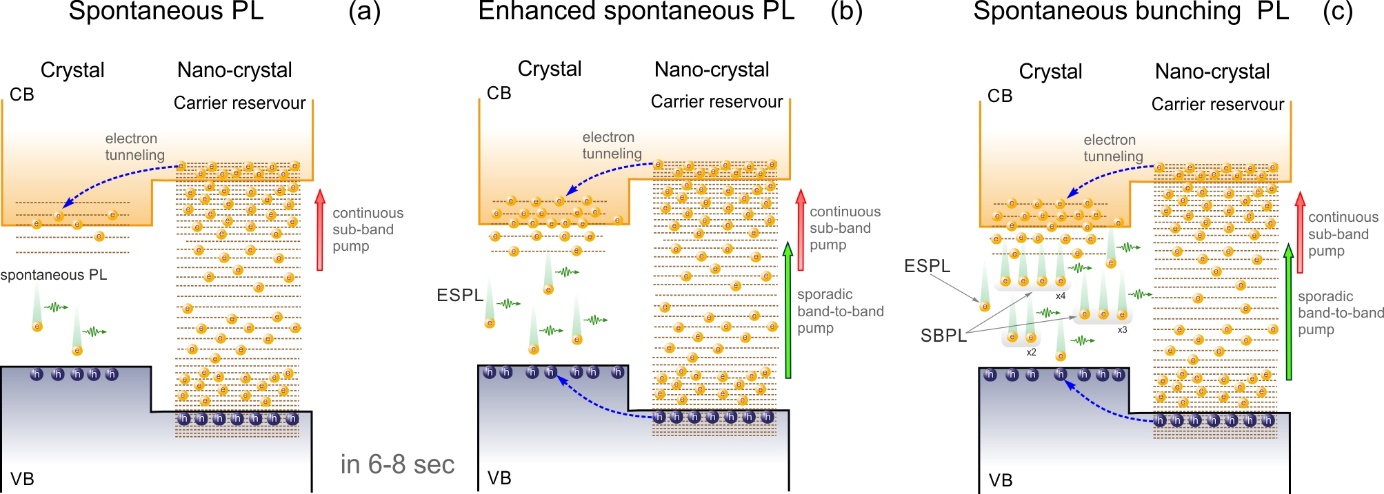


**Figure S11**. Schematic illustration of spontaneous PL (a), enhanced spontaneous PL (b) and spontaneous bunching PL (c) at the disorder-order interface.

**XII. The formation of a polaron at the disorder-order interface**

**
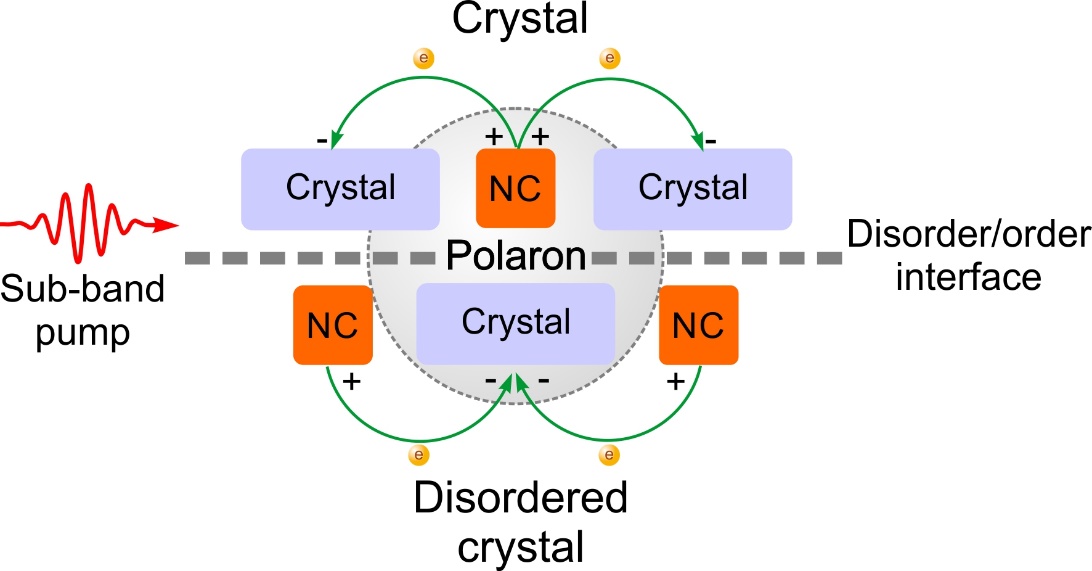
**

**Figure S12**. A physical model of formation of a polaron at the disorder-order interface.

**XIII. Temperature-dependent TERS microscopy of twin domains in CsPbBr_3_**


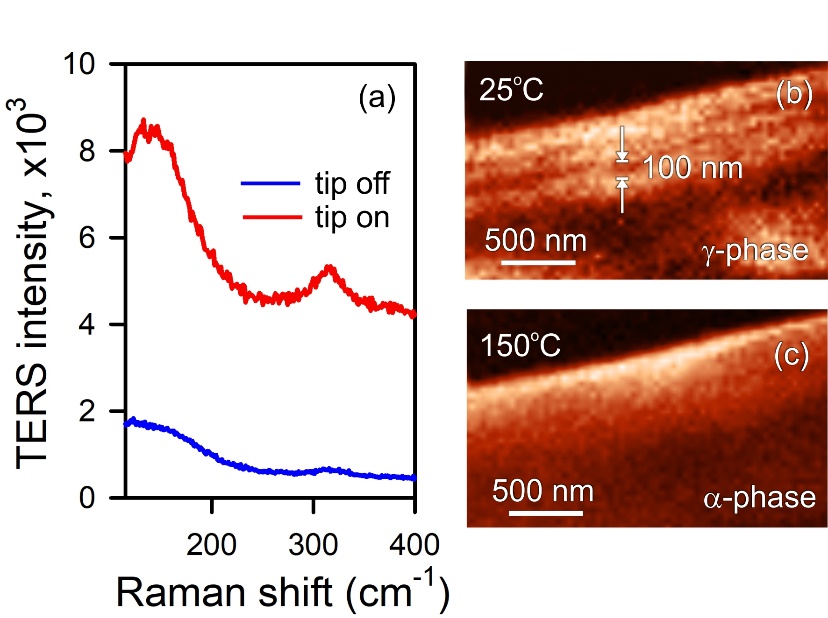


**Figure S13**. (a) Raman spectra of a CsPbBr_3_ crystal when a gold tip off and on at room temperature, (b) and (c) TERS maps of the CsPbBr_3_ crystal at 310 cm^-1^ at temperatures: $25℃$ (γ-phase) and $150℃$ (α-phase).

**XIV. Photon-momentum-enabled inhomogeneous broadening and blueshift upon band-to-band pumping**

**
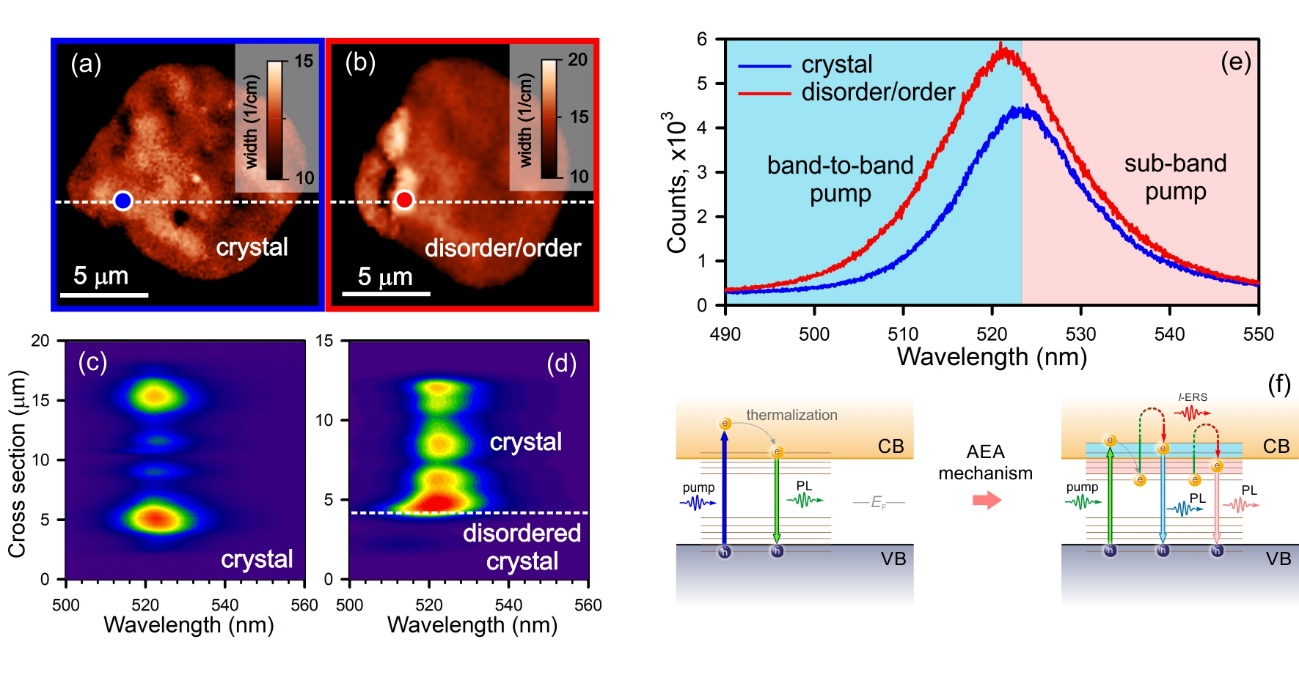
**

**Figure S14**. Linewidth maps for CsPbBr_3_ crystal (a) and disordered/ordered CsPbBr_3_ (b). 1D Raman linewidth maps along the white straight dashed line in Figure S11 a,b for CsPbBr_3_ crystal (c) and CsPbBr_3_ disorder/order (b). (e) Photoluminescence upon 473 nm excitation with the flux of 180 W/cm^2^ at blue and red spots in Figure S11 a,b. (f) Schematic illustration of inhomogeneous broadening and blue-shift through the absorption-emission-absorption (AEA) mechanism.

**XV. *h*-ERS/PL enhancement through all-thermally-induced disordering in CsPbBr_3_**


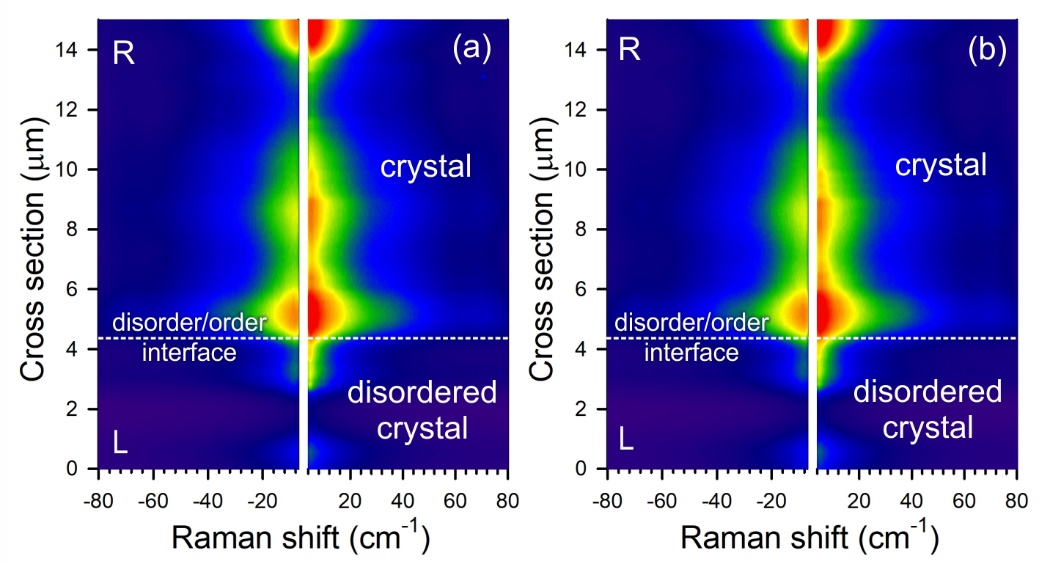


**Figure S15**. 1D *l*-ERS intensity maps of CsPbBr_3_ disordered/ordered crystals
when circuit on (a) and off (b).


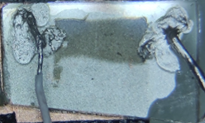


**Figure S16** Optical image of the sample with metallic wires contacting SWNT electrodes via silver paste.
